# A Note upon Repetition

**Published:** 1887-10

**Authors:** 


### A NOTE UPON REPETITION.

Professor Kent says: "The most difficult as well as the most important thing for the homœopath to learn is, how to administer the remedy when selected. In acute cases, when the symptoms are very violent, the action of the remedy is soon overcome by the disease, and it may be necessary to repeat frequently. But as soon as improvement sets in, stop your medicine and give no more till the improvement ceases or other symptoms occur calling for a remedy. Be sure that you have the right remedy the first time. You will save time in most cases, if you are uncertain as to the remedy indicated, to give no medicine till you have returned to your office and consulted your books; for if you administer the wrong remedy it will so mask the case that it will be almost impossible to select the remedy which would have proved curative at first. In chronic diseases, particularly those complicated by psora, in which antipsorics are indicated, it will seldom be necessary to repeat oftener than once in three or four weeks. I very rarely give Sulphur at shorter intervals than four weeks, and some of the most brilliant cures I have ever made were with a single dose of medicine, from which no improvement was manifest for six weeks. From that time improvement was rapid and permanent."—*Medical Era.*

